# Supplementary material for: Efficacy of corticosteroids in non-intensive care unit patients with COVID-19 pneumonia from the New York Metropolitan region
Source: PLoS One. 2020 Sep 9;15(9):e0238827. doi: 10.1371/journal.pone.0238827 (PMC7480842; doi:10.1371/journal.pone.0238827)
Supplement: S1 Table — (DOCX) [file pone.0238827.s001.docx]

**S1 Table. Definitions of variables and outcomes**

| **Variable** | **Definition** |
| --- | --- |
| Index date | Date when corticosteroid is started for corticosteroid group;  Date when PF ratio < 300 or SF ratio < 440 in control group |
| Sofa | SOFA score on index date |
| Spo2 | Spo2 on index date extracted from physician, nursing notes |
| Fio2 | Fio2 for corresponding spo2 |
| Sf | Sp2/fio2 on index date |
| PaO2 | Calculated from SpO2 |
| Pf | Po2/fio2 on index date |
| age | Age of the patient |
| gender | Sex of the patient |
| race | Race of the patient (AA, white, Hispanic, others) |
| corticosteroid | Patient on corticosteroid or not |
| type | Type of corticosteroid (Methylprednisolone, dexamethasone, hydrocortisone, prednisone) |
| dose | Dose of corticosteroid per day |
| dur | Days corticosteroid given |
| Htn | History of Hypertension |
| Dm | History of Diabetes mellitus |
| Copd | History of Chronic obstructive pulmonary disease |
| Cancer | History of Cancer |
| Cad | History of coronary artery disease |
| Hf | History of heart failure |
| QTc | Corrected QT interval in EKG on admission |
| Hb | Hemoglobin on index date |
| Wbc | White blood cell counts on index date |
| Plt | Platelet count on index date |
| Alc | Absolute lymphocyte count on index date |
| creat | Creatinine on index date |
| gfr | Glomerular filtration rate on index date |
| ast | AST on index date |
| alt | ALT on index date |
| pct | Procalcitonin on/before index date |
| LDH | Lactate dehydrogenase on/before index date |
| Ferritin | Ferritin before on/index date |
| Dimer | D-Dimer on/before index date |
| Crp | C-reactive protein on/before index date |
| Il6 | Interleukin-6 on/before index date |
| enoxrx | Enoxaparin treatment dose during the hospital stay |
| tocil | Tociliziumab given or not |
| hcq | Hydroxychloroquine given or not during the hospital stay |
| Icu | Icu admission **since index date** |
| tticu | Date of icu admission |
| Intub | Intubation **since index date** |
| ttintub | Date of intubation |
| dth | Death of patient **since index date** |
| ttdth | Date of patient death |
| primary | Either icu admission or intubation or death outcome **since index date** |
| ttprim | Date of earliest primary outcome |
| los | Length of stay in the hospital |
| dth30 | Death in 30days **since index date** |
| ttdth30 | Time to death within 30 days of index date |
| Sp1 | Spo2 on day1 after index date |
| Fio1 | fio2 on day1 after index date |
| Sf1 | Spo2/fio2 on day1 after index date |
| Spo2 | Spo2 on day2 after index date |
| Fio2 | fio2 on day2 after index date |
| sf2 | Spo2/fio2 on day2 after index date |
| Spo3 | Spo2 on day3 after index date |
| Fio3 | fio2 on day3 after index date |
| Sf3 | Spo2/fio2 on day3 after index date |
| Spo4 | Spo2 on day4 after index date |
| Fio4 | fio2 on day4 after index date |
| Sf4 | Spo2/fio2 on day4 after index date |
| Spo5 | Spo2 on day5 after index date |
| Fio5 | fio2 on day5 after index date |
| Sf5 | Spo2/fio2 on day5 after index date |
