# Supplementary material for: Efficacy of corticosteroids in non-intensive care unit patients with COVID-19 pneumonia from the New York Metropolitan region
Source: PLoS One. 2020 Sep 9;15(9):e0238827. doi: 10.1371/journal.pone.0238827 (PMC7480842; doi:10.1371/journal.pone.0238827)
Supplement: S1 File — (DOCX) [file pone.0238827.s002.docx]

**S1 File. Calculation of SF ratio and PF ratio**

The SF ratio is calculated by SpO_2_ (%) divided by FiO_2_.

FiO2 is estimated for a non-invasive oxygen delivery method in the following way.

1. For nasal cannula - 0.21 for room air, 0.21 + (oxygen flow rate * 0.035)
2. For simple oxygen mask – 0.35 is taken for 6L/min, 0.4 is taken for 7L/min, 0.47 is taken for 8L/min, 0.53 taken for 9L/min, and 0.6 is taken for ≥10L/min^1^
3. For non-rebreather mask – 0.6 is taken for 6L/min, 0.7 is taken for 7L/min, and 0.8 is taken for ≥8L/min ^2,3^

The minimum SF ratio was considered out of multiple SF ratio of the same day.

To calculate the PF ratio, for taken SpO_2_ value, corresponding PaO_2_ value was imputed that is divide by taken FiO_2_. ^2,3^

**References**

1. COVID-19 and O2 therapy. Initial prehospital approach in mild symptomatic patients. | MEDEST. Accessed May 6, 2020. https://medest118.com/2020/03/16/covid-19-and-o2-therapy-initial-prehospital-approach-in-mild-symptomatic-patients/

2. Brown SM, Duggal A, Hou PC, et al. Nonlinear Imputation of PaO2/FIO2 From SpO2/FIO2 Among Mechanically Ventilated Patients in the ICU. *Crit Care Med*. 2017;45(8):1317-1324. doi:10.1097/CCM.0000000000002514

3. Brown SM, Grissom CK, Moss M, et al. Nonlinear Imputation of PaO2/FIO2 From SpO2/FIO2 Among Patients With Acute Respiratory Distress Syndrome. *Chest*. 2016;150(2):307-313. doi:10.1016/j.chest.2016.01.003
