# Supplementary material for: Efficacy of corticosteroids in non-intensive care unit patients with COVID-19 pneumonia from the New York Metropolitan region
Source: PLoS One. 2020 Sep 9;15(9):e0238827. doi: 10.1371/journal.pone.0238827 (PMC7480842; doi:10.1371/journal.pone.0238827)
Supplement: S2 File — (DOCX) [file pone.0238827.s003.docx]

**S2 File. Multivariate Cox-Proportional Model Building**

The final model was built by both the Forward selection and backward elimination method. P-value 0.15 was set as a cut-off for the variable to include in the final multivariable model. The variables mentioned in the literature that can possibly be associated with the outcomes, were also included in the final model. In the final model, SF ratio, age, gender, COPD, WBC, platelet count, tocilizumab, and therapeutic dose of Enoxaparin were included for adjustment. In the setting of missing values for D-dimer, C-reactive protein, AST, ALT variables were not included in the final multivariable model.
