# Supplementary figures and images for: Efficacy of corticosteroids in non-intensive care unit patients with COVID-19 pneumonia from the New York Metropolitan region
Source: PLoS One. 2020 Sep 9;15(9):e0238827. doi: 10.1371/journal.pone.0238827 (PMC7480842; doi:10.1371/journal.pone.0238827)

**S1 Fig. Kaplan-Meier curves for Intensive care unit transfer**


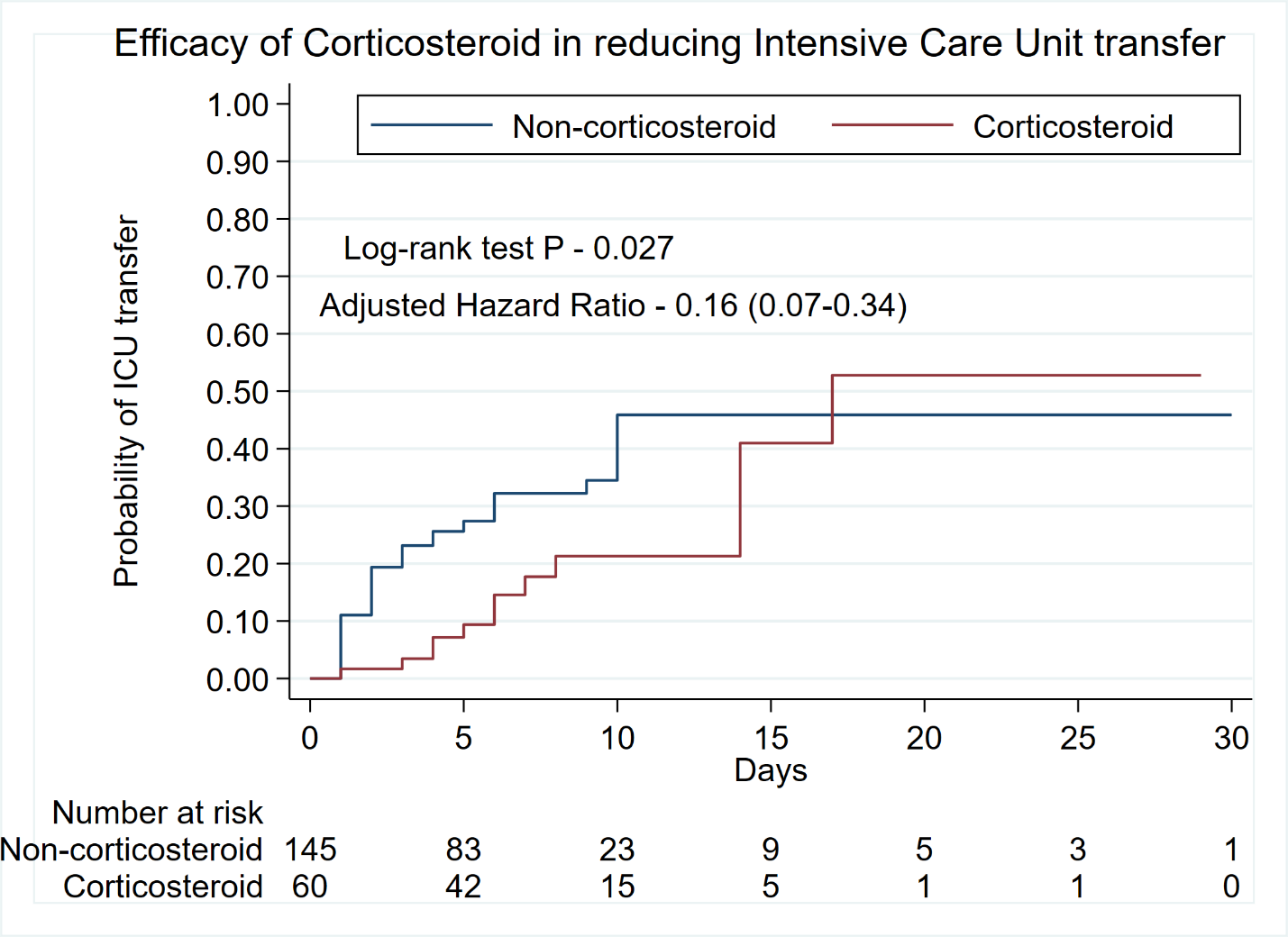

Supplement: S1 Fig — (DOCX) [file pone.0238827.s004.docx]

**S2 Fig. Kaplan-Meier curves for Intubation**


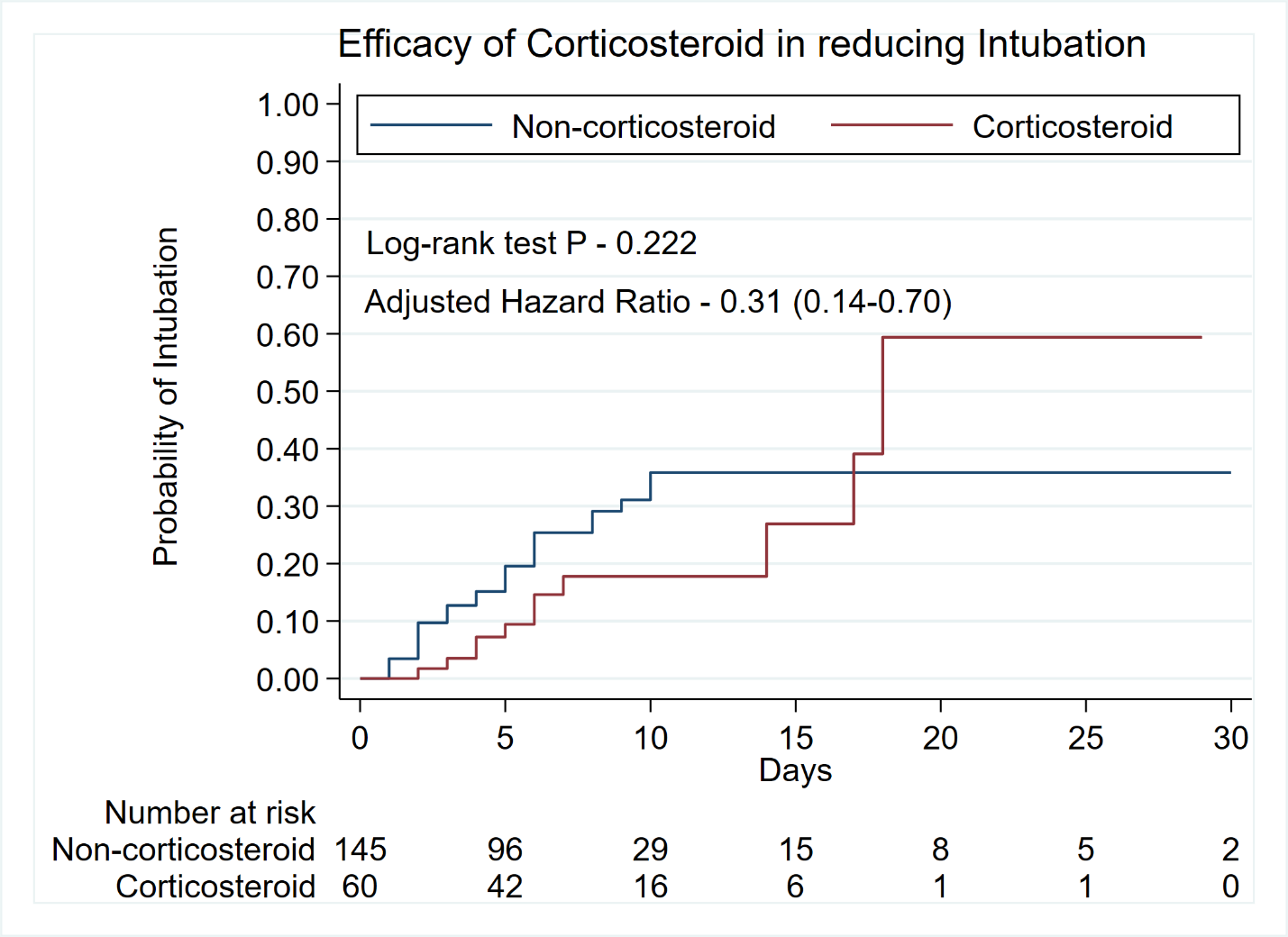

Supplement: S2 Fig — (DOCX) [file pone.0238827.s005.docx]

**S3 Fig. Kaplan-Meier curves for Discharge**

**
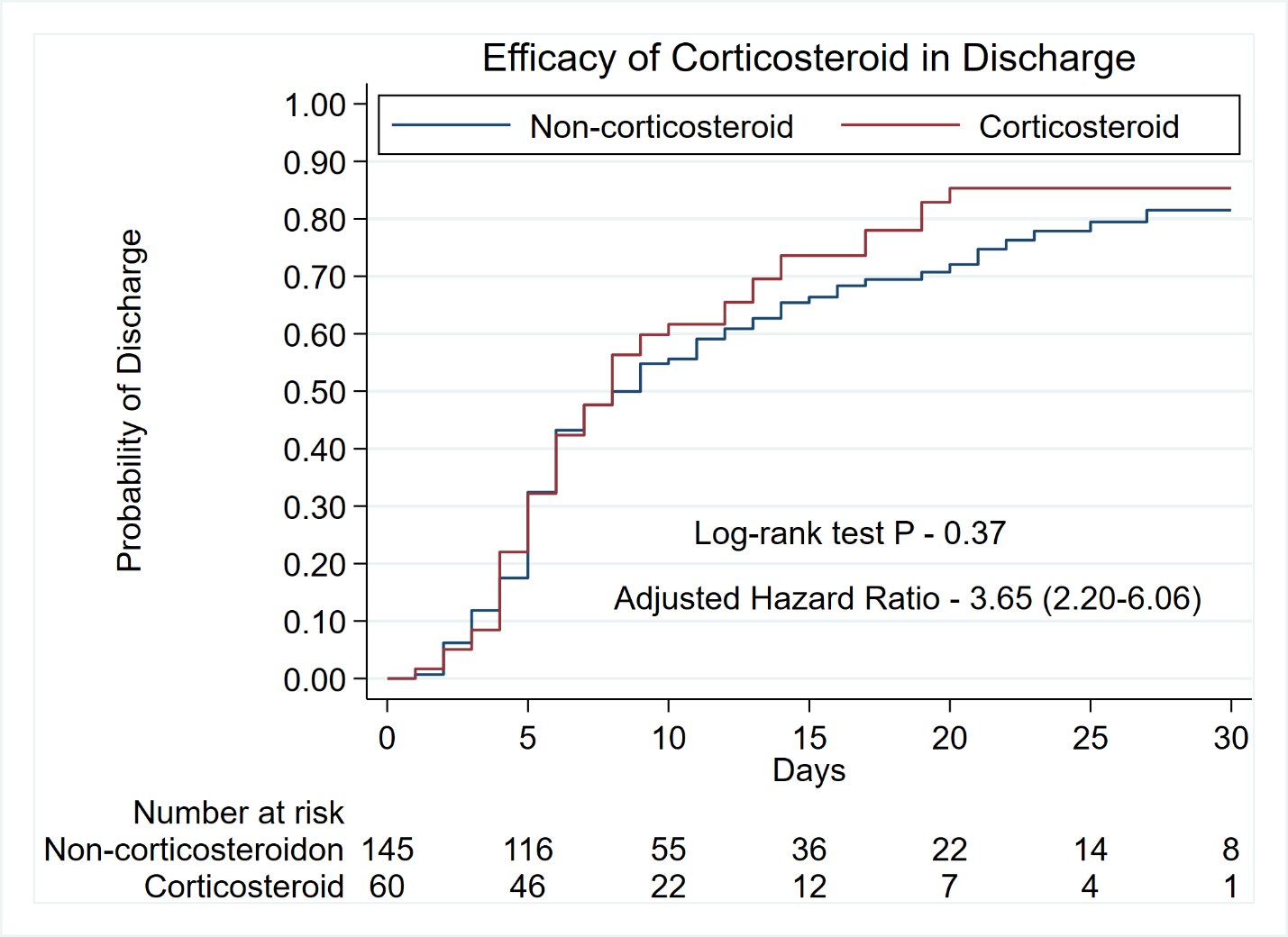
**

Supplement: S3 Fig — (DOCX) [file pone.0238827.s006.docx]
